# Datasets of land use change and flood dynamics in the vietnamese mekong delta

**DOI:** 10.1016/j.dib.2022.108268

**Published:** 2022-05-15

**Authors:** Hoang Thai Duong Vu, Huu Long Vu, Peter Oberle, Schenk Andreas, Phuc Canh Nguyen, Dung Duc Tran

**Affiliations:** a Karlsruhe Institute of Technology, Karlsruhe 76131, Germany; b School of Public Finance, College of Economics, Law and Government, University of Economics, Ho Chi Minh City, Viet Nam; c Center of Water Management and Climate Change, Institute for Environment and Resources, Vietnam National University – Ho Chi Minh city (VNU – HCM), Ho Chi Minh City, Viet Nam

**Keywords:** Data, Flood, Land use, MODIS products, Vietnamese Mekong delta

## Abstract

This paper compiles the data associated with a research article published in STOTEN [1]. The data set represents figures, tables, and images illustrating the temporal and spatial distribution of land use and flood dynamics from 2000 to 2020 in the Vietnamese Mekong Delta (VMD). The MODIS imageries were freely accessed online via the NASA website [2] and processed to land use and flood maps based on the algorithms by Sakamoto et al. [3,4]. The MODIS products show a high validation with statistical data and radar satellites [1]. The datasets of flood map and land use, therefore, are available to scientists, engineers, and policy-makers in agricultural management associated with flood management in the VMD. They could be used for policy settings, household livelihood assessment as well as other economic analyses for the VMD region due to the change of land use and flooding dynamics.

## Specifications Table

**Table:** 

Subject	Agriculture, Environmental Science
Specific subject area	Analyzing land use change and flooding dynamics in the Vietnamese Mekong Delta (VMD) for a period from 2000 to 2020 to monitor holistically the land use change and flooding situation in the region.
Type of data	Table Map
How data were acquired	MODIS imageries were downloaded freely from the NASA website and processed manually to land use and flood maps based on ENVI and GIS programs. Statistical data of rice and aquaculture areas were accessed via the GSO website to be calibrated with MODIS land use data.
Data format	Analyzed flooding maps (figure, geotiff) Analyzed land use maps (figure, geotiff, excel sheet)
Parameters for data collection	MODIS products were processed into flooding and land use data as described in Section 2.
Description of data collection	The Moderate-resolution Imaging Spectro-radiometer (MODIS) was selected as the most suitable satellite to serve for this study. Herein, the MODIS products of MOD09A1 (Terra Surface Reflectance 8-Day Global 500m) are applied to process the maps of land use and flood extension. The imageries have a spatial resolution of 500m and were freely downloaded at the NASA website [2]. Statistical data is collected in this study to validate the satellite land use maps as well as to examine the effect of rice production due to land use change. The areas of rice and aquaculture were collected online from 2000 to 2020 at the General Statistics Office of the Vietnam website [5].
Data source location	Region: Vietnamese Mekong Delta Country: Vietnam Latitude: 8.5°N – 11.5°N Longitude: 104°E – 107°E Primary data sources: MODIS products: https://doi.org/10.5067/MODIS/MOD09A1.006 Statistical data: General Statistical Office (GSO), Vietnam https://www.gso.gov.vn/en/agriculture-forestry-and-fishery/.
Data accessibility	Repository name: Datasets of land use and flood dynamics in the Vietnamese Mekong Delta. Data identification number (doi): 10.17632/kpftzmsyyz.2 Direct URL to data: https://data.mendeley.com/datasets/kpftzmsyyz/2
Related research article	H.T.D. Vu, D.D. Tran, A. Schenk, C.P. Nguyen, H.L. Vu, P. Oberle, T.C. Van, F. Nestmann, Land use change in the Vietnamese Mekong Delta: New evidence from remote sensing. The Science of the Total Environment, p. 151918 (2021). DOI: 10.1016/j.scitotenv.2021.151918.

## Value of the Data

• We contribute a continuous dataset of land use/land cover (21 maps) and flood dynamics (567 maps) from 2000 to 2020 with detailed spatial data. Hence, they are useful for the VMD agricultural and aquaculture management plan integrated with flood management in the VMD.
• Scientists, administrators, decision-makers, and engineers can benefit from these datasets.
• Flooding data can be used/reused for flood risk management in the VMD, i.e., construction dike planning, calibration of flood extension for hydraulic models [6], [7], planning of residential areas, and rice development. Besides, a comparison of flooding management between the countries in the lower Mekong River Basin (i.e., Cambodia and Vietnam) could be evaluated based on the MODIS flooding maps, which cover the territories of Cambodia and Vietnamese Mekong Delta.
• The dataset including 567 flooding maps from 2000 to 2020 is a valuable database to setup a flood monitoring website portal for the VMD region.
• In addition, the MODIS land use and flooding products can be used for cross-validation with other satellite products with higher spatial resolutions, i.e., Sentinel, Landsat, Copernicus, etc. to assess the appropriateness and accuracy of land use/land cover and flood maps based on remote sensing.
• Last but not least, MODIS land use maps are important for agricultural and aquaculture management plans. They could be a valuable input for (i) evaluating the household livelihoods of local farmers in the last two decades, (ii) economic analyses of cost and benefits, (iii) assessment of agricultural efficiency and rice productivity due to the conversion of rice cropping patterns from double rice to triple rice.

## 1 Data Description

**Fig. 1:**
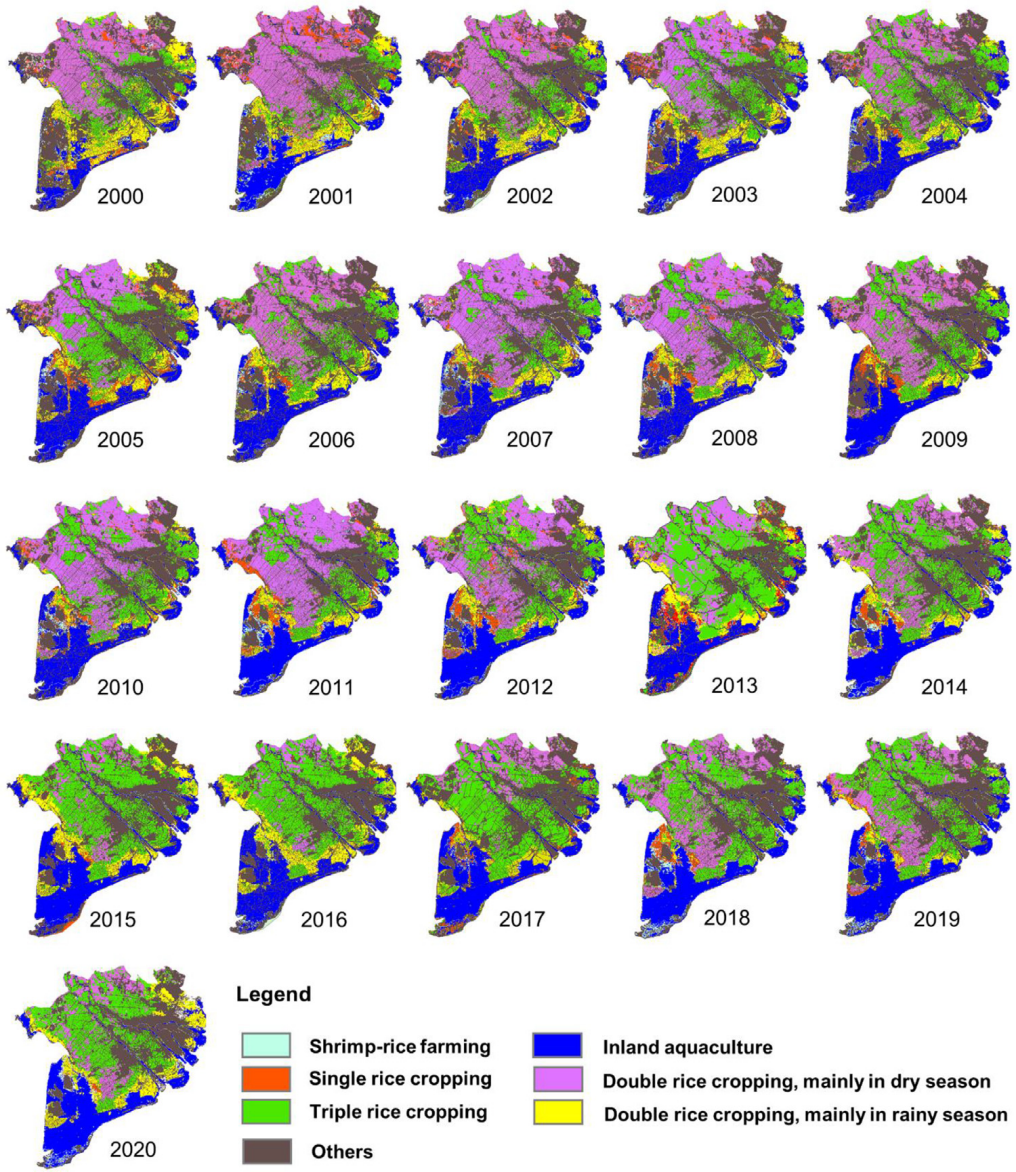
Land use change in the VMD from 2000 to 2020.

The data of land use change in the VMD are presented in Fig. 1, Tables 1 and 2. Particularly, Fig. 1 shows a temporal and spatial view of land use time series from 2000 to 2020 with seven objects of shrimp-rice farming (cyan), inland aquaculture (blue), single rice (orange), double rice type 1 - mainly in the dry season (violet), double rice type 2 - mainly in the rainy season (yellow), and other types of land use (brown), i.e., built-up areas, infrastructures, forests, orchards, and mixture pixels.

**Table 1:** Total rice-planted area in the VMD.

a) Total rice area based on MODIS satellite (thous. ha)																					
Name	2000	2001	2002	2003	2004	2005	2006	2007	2008	2009	2010	2011	2012	2013	2014	2015	2016	2017	2018	2019	2020
Long An	511	497	460	501	469	493	446	510	530	471	571	568	600	509	486	539	571	484	522	488	490
An Giang	486	485	513	541	559	564	555	553	579	592	609	617	622	696	657	663	682	688	615	672	647
Tien Giang	274	274	238	274	256	251	245	243	244	244	249	232	232	217	239	233	241	211	201	200	173
Vinh Long	185	170	179	188	178	194	179	157	177	176	170	166	165	202	182	182	184	199	162	160	154
Kien Giang	539	578	560	557	599	562	609	539	584	624	613	603	660	614	705	759	759	676	720	656	747
Ben Tre	78	98	70	74	67	66	73	77	74	78	82	78	82	59	73	60	47	68	52	51	34
Can Tho	406	376	358	394	212	215	189	165	172	174	169	162	153	246	192	227	228	251	237	209	204
Hau Giang^1^	-	-	-	-	185	224	199	183	174	197	220	186	171	188	202	211	220	184	195	168	198
Tra Vinh	182	198	205	200	197	169	187	191	197	203	216	211	192	265	225	220	193	261	223	212	181
Bac Lieu	208	157	143	121	129	116	155	135	152	170	142	174	152	170	162	161	154	175	185	170	158
Soc Trang	342	364	354	326	344	317	361	327	340	367	370	369	373	394	368	378	352	403	352	356	326
Ca Mau	224	96	83	61	48	40	50	22	53	51	55	64	56	76	74	76	74	131	117	64	66
Dong Thap	390	459	490	515	540	552	531	491	492	548	526	513	587	579	618	632	628	549	520	617	584
Mekong delta	3824	3752	3653	3752	3783	3764	3777	3592	3769	3896	3993	3944	4045	4215	4183	4342	4332	4279	4102	4023	3963


b) Total rice-planted area based on statistical data (thous. ha)																					
Name	2000	2001	2002	2003	2004	2005	2006	2007	2008	2009	2010	2011	2012	2013	2014	2015	2016	2017	2018	2019	2020
Long An	453	441	433	424	433	429	433	428	457	464	471	484	500	528	519	523	527	527	511	506	503
An Giang	464	459	477	504	523	530	504	520	565	557	587	608	625	641	626	644	669	641	623	626	637
Tien Giang	282	276	265	261	259	252	248	247	245	246	244	242	241	236	231	225	216	211	201	184	136
Vinh Long	209	216	210	207	208	203	197	158	177	177	170	182	186	182	180	181	176	169	162	155	146
Kien Giang	541	551	576	563	570	596	595	583	609	622	643	687	725	770	754	770	766	735	728	722	726
Ben tre	102	101	100	96	91	84	82	80	79	81	80	77	76	72	67	63	42	55	52	48	22
Can Tho	413	441	457	453	230	232	223	208	219	209	209	225	228	237	232	238	240	240	237	225	223
Hau Giang^1^	-	-	-	-	228	228	227	189	203	191	211	213	214	212	205	207	202	207	195	196	198
Tra Vinh	237	240	236	236	236	232	228	224	227	232	233	233	227	236	236	236	211	220	223	224	205
Bac Lieu	217	178	170	150	137	141	144	150	155	167	158	164	179	182	180	181	172	181	185	189	188
Soc Trang	370	349	355	350	315	322	324	325	322	335	350	349	366	374	364	363	357	348	352	356	354
Ca Mau	248	132	131	107	132	110	115	123	133	139	126	130	129	130	127	127	112	113	117	116	112
Dong Thap	408	408	426	436	453	468	454	447	468	451	465	501	488	542	529	546	551	538	520	522	514
Mekong delta	3946	3792	3835	3787	3816	3826	3774	3683	3859	3870	3946	4094	4184	4340	4250	4302	4241	4185	4108	4070	3964

1 Note: Hau Giang province was separated from Can Tho province since 2004.

**Table 2:** Inland aquaculture area in the VMD.

a) Aquaculture area based on MODIS satellite (thous. ha)																					
Name	2000	2001	2002	2003	2004	2005	2006	2007	2008	2009	2010	2011	2012	2013	2014	2015	2016	2017	2018	2019	2020
Long An	13.1	18.6	18.4	21.7	16.7	20.2	19.9	16.7	28.6	18.4	16.0	23.8	16.3	11.7	17.9	14.8	14.2	9.5	16	13.3	12.5
An Giang	8.3	3.9	3.3	5.6	2.6	3.7	3.0	3.1	7.7	3.5	2.7	4.6	4.9	7.4	5.5	6.8	5.3	3.5	5	5.2	4.8
Tien Giang	11.1	9.5	10.1	12.0	10.2	9.8	11.1	9.3	12.5	12.1	8.2	11.9	11.1	8.0	13.7	13.7	12.1	10.1	13	11.9	12.4
Vinh Long	5.1	2.6	3.1	4.7	3.0	3.0	3.4	3.4	5.5	2.9	3.4	4.2	3.4	4.5	4.7	5.5	4.4	3.3	4	3.5	3.2
Kien Giang	34.2	39.0	47.8	63.8	60.7	66.8	60.4	75.8	71.3	59.9	70.1	72.6	66.9	69.3	93.7	108.2	103.7	109.0	100	118.8	132.6
Ben Tre	42.0	48.0	45.1	53.0	49.8	49.7	49.6	48.2	53.1	53.2	43.6	56.3	53.3	40.8	59.3	56.3	48.4	45.9	58	52.6	54.4
Can Tho	1.9	1.6	2.4	2.6	2.1	2.3	2.2	2.4	4.1	2.4	2.3	3.9	3.0	3.4	3.0	3.4	3.2	2.5	3	3.2	2.6
Hau Giang^1^	-	-	-	-	0.1	0.2	0.1	0.1	0.4	0.1	0.2	0.4	0.3	0.2	0.4	0.3	0.2	0.2	0	0.4	0.3
Tra Vinh	29.1	38.3	35.9	42.2	42.4	42.1	44.5	41.1	46.0	45.6	39.7	49.3	47.0	38.7	51.5	48.8	44.5	45.4	56	50.0	51.6
Bac Lieu	57.4	114.0	102.9	116.6	116.2	118.1	112.9	118.4	111.4	128.7	116.4	139.2	135.3	112.9	137.6	143.8	118.9	148.4	141	139.8	141.4
Soc Trang	32.1	51.7	45.3	60.2	57.0	60.3	58.2	57.2	58.6	58.6	55.8	70.3	63.7	52.5	71.5	65.9	59.1	67.1	70	68.2	70.1
Ca Mau	162.0	286.0	255.4	272.7	273.3	282.3	275.8	295.5	280.8	316.5	280.7	344.5	345.9	275.1	350.7	332.3	291.6	304.6	343	344.5	362.9
Dong Thap	8.0	5.4	4.3	9.4	4.6	5.1	3.9	4.5	10.3	4.6	3.0	8.8	6.6	9.0	9.2	9.6	6.7	6.2	8	8.2	4.6
Mekong delta	404.2	618.6	573.8	664.4	638.6	663.4	644.9	675.6	690.2	706.5	641.9	789.6	757.5	633.4	818.6	809.2	712.3	755.5	816.4	819.4	853.2


b) Aquaculture area based on statistical data (thous. ha)																					
Name	2000	2001	2002	2003	2004	2005	2006	2007	2008	2009	2010	2011	2012	2013	2014	2015	2016	2017	2018	2019	2020
Long An	3.4	6.6	7.3	10.2	12.4	13.2	11.6	12.6	10	9	9.4	10.8	8.9	9	8.7	8.7	8.2	9.4	11.1	10.3	10.1
An Giang	1.3	1.3	1.8	1.6	1.9	1.8	1.9	3	2.8	2.5	2.4	1.8	1.8	2.5	2.4	2.5	2.5	2.7	3.3	3.5	3.3
Tien Giang	8.4	8.8	9.6	10.8	11.9	12.1	12.4	12.9	12.6	12.6	13.1	14.1	14.4	15.4	15.7	12.6	15.8	15.2	15.1	15.9	14.9
Vinh Long	1.4	1.3	1.4	1.5	1.6	1.8	2.3	2.3	2.4	2.5	2.4	2.5	2.4	2.6	2.4	2.4	2.4	2.3	2.4	2.6	2.5
Kien Giang	34.6	42.6	49.7	62.1	79.2	82.2	95.5	106.2	134.6	121.7	123.1	114.6	115.5	126.9	132.9	136.2	142.7	153.9	160.7	166.5	171.5
Ben tre	29.3	25.6	36	37.7	41.1	42.3	41	41.9	42.1	42	42.5	43.1	47.7	44.8	47.1	42.4	45.2	45.2	45.4	45.9	38
Can Tho	12.6	13.6	16.5	10	11	12.5	13.6	14	12.9	13.1	12.8	12.6	11.7	11	11.4	10.9	8.4	8.3	7.6	7.1	7.2
Hau Giang^1^	-	-	-	-	8.3	8.9	7.4	8.4	6.1	6.2	6.4	6.4	6.6	6.5	7.1	6.8	7.1	7.3	7.4	7.8	8.1
Tra Vinh	52.6	54.3	25.2	30.2	32.5	38.7	41.3	42.5	36.4	34	32.8	29.1	40.4	36.9	30.8	29.5	30.4	32.4	32.5	36	41.5
Bac Lieu	54	83	100.6	112.3	118.8	118.7	120.2	122.2	125.6	126.3	125.4	125.2	117.8	127.9	127.5	130.6	131.8	136.1	138.9	140.5	140.5
Soc Trang	41.4	53.2	48.3	57.1	59	64.9	64.3	62	67.7	69.2	71.5	67.1	64.8	68.2	68.4	68.8	69.5	74.1	77.9	78.9	76.3
Ca Mau	204.4	254.2	271.4	277.7	277.7	279.2	275.2	290.8	293.2	294.7	296.1	296.5	296.5	295.8	298.1	299.8	301.5	302.9	302.4	305	285.5
Dong Thap	1.9	2.3	2.6	2.6	3.2	3.6	4.5	5	5.8	5	4.8	5.5	5.7	5.9	6	5.8	5.8	6.2	6.3	6.5	6.4
Mekong delta	445.3	546.8	570.4	613.8	658.6	679.9	691.2	723.8	752.2	738.8	742.7	729.3	734.2	753.4	758.5	757.0	771.3	796.0	811.0	826.5	805.8

1 Note: Hau Giang province was separated from Can Tho province since 2004.

**Fig. 2:**
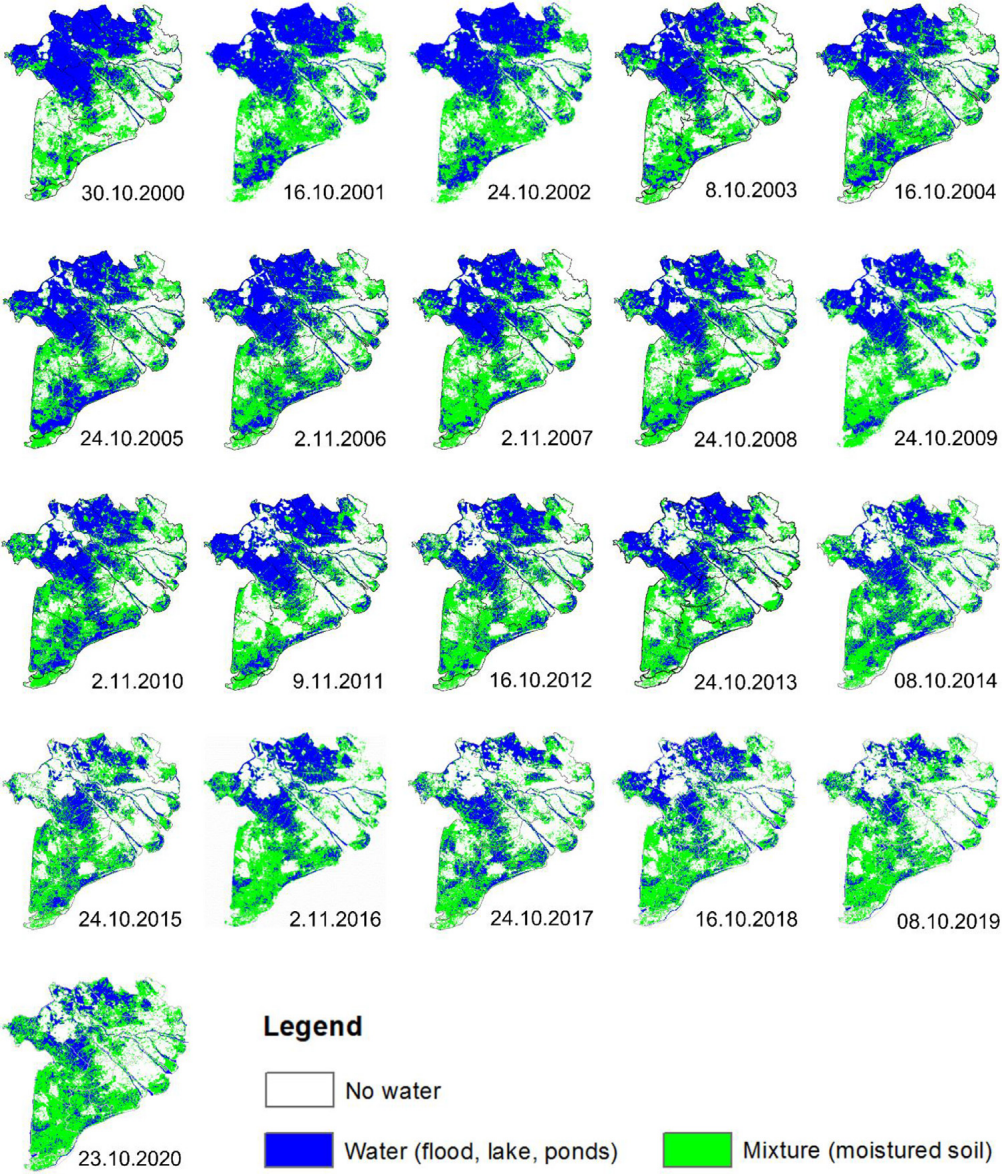
Maximum flood extension in the VMD from 2000 to 2020.

Tables 1 and 2 show the values of total rice planted area and total aquaculture area between statistical data and MODIS products in the VMD from 2000 to 2020. In general, a good agreement between statisital data and the MODIS-derived estimation areas can be reached with a correlation R^2^ > 0.96 over the whole VMD [1]. However, in some cases the MODIS data shows large differences in comparison with statistical data, e.g. in Tra Vinh or Ca Mau provinces. This estimation error was addressed by Sakamoto et al. [4] due to the mixed-pixel effect of the MODIS sensor with the moderate resolution of 500 m. Excluding the mixed-pixel effect in the MODIS-derived estimates could give good agreement between the obtained spatial patterns of the multiple rice cropping systems and regional land use data [4]. On the other hand, the statistical data books do not have spatial information on land use and land cover, therefore the MODIS-derived land use data contributes to a much better understanding of the spatial distribution of farming systems and annually change of land use patterns in the VMD [1].

The total rice planted area and aquaculture area are annually calculated as:

• Total rice planted area = Single rice + (Double rice type 1 + Double rice type 2) x 2 + Triple rice x 3
• Total aquaculture area = Shrimp-rice farming area + Inland aquaculture area

For more information on the land use data estimated from MODIS products, please find the excel sheet “Land use data.xlsx” in the Supplementary Materials.

Besides, Fig. 2 illustrates the maximum flooding extension in the VMD. The flooding maps contain three main classes with water (blue), mixed pixel (green), and non-flooded areas (white). Here, the maximum flooding extension is determined as the date with the largest flooding extent in the VMD, which occurs annually between October 8th and November 9th [1].

Overall, the analyzed data of flooding and land use are published online for further assessment. The data can be downloaded at https://data.mendeley.com/datasets/kpftzmsyyz/2. Where:

• Flooding data are displayed in GeoTiff format in “Flooding” folder and named “Flood_year_DOY”. Here, DOY indicates Day of the Year, and the flooding maps reach annually from early June (Flood_year_153) to end of December (Flood_year_361). The definition of flooding maps is presented in Table 3.

**Table 3:** Classes of flooding maps.

No.	Value	Description	Color code (R-G-B)
1	1	Water (flooding, lake, ponds)	0 - 0 - 255
2	2	Mixture	0 - 255 – 0
3	0	No water (or no data due to out of the study area)
• Land use data set are GeoTiff files in “Land use” folder and named “Land use_year”. In which, “year” indicates from 2000 to 2020. The definition of land use maps is displayed in Table 4.

**Table 4:** Classes of land use maps.

No.	Value	Description	Color code (R-G-B)
1	1	Inland aquaculture	0 - 0 - 255
2	2	Shrimp-rice farming	190 - 255 - 232
3	5	Others (built-up areas, infrastructures, forests, orchards, and mixtures pixels)	100 - 78 - 78
4	6	Single rice cropping	255 - 85 - 0
5	7	Triple rice cropping	76 - 230 - 0
6	8	Double rice cropping – mainly in the dry season	223 - 115 - 255
7	9	Double rice cropping – mainly in the rainy season	255 - 255 - 0

## 2 Experimental Design, Materials and Methods

The imageries of MODIS products were processed for flood mapping and land use detection in the VMD according to algorithms developed by Sakamoto et al. [3] and Sakamoto et al. [4]. The approaches of flood mapping and land use detection apply three important indexes, i.e., Enhanced Vegetation Index (EVI), Land Surface Water Index (LSWI), and Difference Value between EVI and LSWI (DVEL), which are expressed as:

(1) $$EVI=2.5\frac{NIR-RED}{NIR+6RED-7.5BLUE+1}$$

(2) $$LSWI=\frac{NIR+SWIR}{NIR+SWIR}$$

(3) DVEL=EVI−LSWI

where RED is the red band (sur_refl_b01), NIR is the near-infrared band (sur_refl_b02), SIWR is the short-wave infrared band (sur_refl_b06), and BLUE is the blue band (sur_refl_b03) of the MODIS surface reflectance.

The flood maps were processed into three classes including (i) Flood; (ii) Mixed pixel; and (iii) No water (see Table 3). While the output of the land use maps contains eleven classes Vu et al. [1]. In this paper, we grouped the classes of land use maps into seven objects to be more convenient for users, see Table 4. Hence, the land use maps include shrimp-rice farming, inland aquaculture, single rice, double rice cropping in the dry season, double rice cropping in the rainy season, triple rice cropping, and others (forest, orchard, extensive farming, mixtures in flood-prone areas, mixtures in double rice and triple rice cropping, and unused areas).

A detailed description of flood and land use processing could be referred to Vu et al. [1], Sakamoto et al [3]; Sakamoto et al. [4], and/or Vu [8].

## CRediT Author Statement

**Hoang Thai Duong Vu:** Conceptualization, Methodology, Software, Data curation, Writing - Original draft preparation, Writing - Reviewing and Editing; **Huu Long Vu:** Software, Data curation, Writing - Original draft preparation, Writing - Reviewing and Editing; **Peter Oberle:** Methodology, Visualization, Writing - Reviewing and Editing; **Andreas Schenk:** Methodology, Software, Visualization, Project administration, Writing - Reviewing and Editing; **Phuc Canh Nguyen:** Writing - Original draft preparation, Writing - Reviewing and Editing; **Dung Duc Tran:** Writing - Original draft preparation, Writing - Reviewing and Editing.

## Ethics Statement

This work does not involve chemicals, procedures or equipment that have any unusual hazards inherent in their use. This work does not involve the use of animal or human subjects.

## Declaration of Competing Interest

The authors declare that they have no known competing financial interests or personal relationships which have or could be perceived to have influenced the work reported in this article.

## Data Availability

Datasets of Land Use Change and Flood Dynamics in the Vietnamese Mekong Delta (Original data) (Mendeley Data).

## Appendix Supplementary materials

Image, application 1
